# Integrated multi-population selection-signature scans identify putative functional genes influencing litter size in jishen black pigs

**DOI:** 10.1186/s12864-026-12960-z

**Published:** 2026-06-29

**Authors:** Changyi Chen, Xiaoran Zhang, Jing Li, Juan Ke, Hao Sun, Chunyan Bai, Boxing Sun

**Affiliations:** College of Animal Science, Jilin University, Changchun, 130062 China

**Keywords:** Jishen Black pig, Litter size, Population genetics, *KCNQ1*, Selection signature

## Abstract

**Background:**

Reproduction traits constitute the primary objective of porcine genetic improvement programs, with litter size being the principal determinant of herd reproduction output. Relative to the intensively selected Large White line, as a breed derived from Large White (LW) and Beijing Black (BJB) pigs, Jishen Black (JSB) pigs exhibit markedly lower litter size, indicating substantial potential for genetic improvement.

**Results:**

Whole-genome resequencing data were obtained from 110 individuals of six pig populations differed in litter size: a Large White line with high litter size (> 16, LWH), cryopreserved Large White samples from 1977 (LW1977), dam-selected (LWD) and sire-selected (LWS) lines derived from LW1977, Berkshire (BKS), and Jishen Black pigs (JSB). Principal component analysis (PCA) captured clear population genetic structure along PC2, with the observed distribution pattern descriptively coinciding with litter size variation across populations, though such an association was not statistically verified. A composite selection-signature scan that integrated *Fst*, π-ratio, and XP-EHH was performed between multiple population groups. Candidate signals were filtered by excluding those also detected in a control comparison (Jishen Black vs. Berkshire) that showed minimal litter‑size difference, to screen selection signatures with potential relevance to reproductive trait differentiation at the population level. Twelve genes were identified as putative candidate genes for litter size, including *PEX14*, *CDK15*, *KCNQ1*, *SPAG17*, *TTF2*, *CD101*, *CASQ2*, *VANGL1*, *ARID5B*, *KLHL32*, *EML1*, and *NAV1*. Functional enrichment analysis indicated that these genes significantly over-represented in microtubule-related biological processes. Among them, *KCNQ1* was consistently detected by all methods and comparison groups. Further analysis of *KCNQ1* revealed multiple SNPs with significant allele-frequency differentiation among populations; notably, two intronic variants (chr2:A1,861,604G and chr2:A1,867,076G) showed population-specific allele frequency patterns that descriptively aligned with the stratification of litter size performance.

**Conclusions:**

By comprehensively dissecting population genomic differentiation among multi-breed populations divergent in litter-size performance, this study implemented established a feasible framework for screening candidate loci associated with porcine prolificacy at the population level, expanded the gene list with suggestive selection signatures, and provided potential molecular markers requiring further independent cohort validation and functional verification to support future genetic improvement in swine reproduction efficiency.

**Supplementary Information:**

The online version contains supplementary material available at https://doi.org/10.1186/s12864-026-12960-z.

## Introduction

Reproduction traits constitute the primary selection objectives in porcine genetic improvement programs, among which total number born and number of live born piglets are the proximate determinants of herd-level reproduction efficiency. Genetic progress in these traits elevates annual piglets-weaned per sow and simultaneously lowers unit production costs through the dilution of fixed inputs, conferring pivotal leverage on both biological efficiency and economic profitability [1]. However, the heritability of reproduction traits in pigs is generally low [2], notwithstanding decades of quantitative trait locus (QTL) mapping and candidate-gene surveys, mutations of demonstrable causality or major effect loci amenable to routine genomic selection remain elusive [3]. Moreover, marked divergence in genetic background, local adaptation, and selection history among regional pig populations translates into substantial inter-breed variation in reproduction output. Consequently, current efforts are increasingly devoted to dissecting the genetic architecture of this phenotypic diversity by pinpointing breed-specific variants that modulate reproduction capacity [4]. Therefore, systematically elucidating the genetic architecture of each pig herd and precisely characterizing their reproduction traits are prerequisites and key factors for achieving targeted breeding of high-fertility breeds.

Whole-genome resequencing now serves as a high-resolution platform for dissecting genetic diversity and selection signature in swine [5–7]. Genome-wide scans at the population level have systematically delineated an extensive repertoire of candidate loci and functional variants underlying adaptive responses, phenotypic plasticity and economically relevant traits, thereby furnishing critical marker repositories for genomic selection [7]. Leveraging resequencing datasets from high-versus low-prolific Yorkshire sows, Li et al. [8] deployed fixation index (*Fst*) and cross population extended haplotype homozygosity (XP-EHH) metrics to detect selection footprints, nominating *NEDD9*, *SLC39A11*, *SNCA* and *UNC5D* as candidate regulators of reproduction performance. Wang et al. [9] integrated LD (linkage-disequilibrium) decay with selection-signature analyses to pinpoint *CCNB2*, *TRPM6* and *EYA* as loci implicated in folliculogenesis and embryonic survival in Xiang pigs. Wang et al. [10] used Tongcheng pigs as a model and employed genome-wide selection-signature mining to reveal genomic imprints of artificial selection on reproduction traits. In summary, a multidimensional resequencing analysis strategy can systematically elucidate the genetic architecture of pig reproduction traits and efficiently identify causal variants and functional genes influencing litter performance.

The Jishen Black (JSB) pig is a synthetic breed developed through nearly three decades of selective breeding following the initial crossbreeding between Large White and Beijing Black (BJB) pigs. The BJB itself is a composite lineage derived from multiple ancestral breeds, including Chinese Northern indigenous breeds, Huanghuaihai pigs, Large White, and Berkshire, among others [11]. Subsequent multi-breed crossing has further augmented the genetic diversity of JSB. Despite prolonged selection efforts, the reproduction performance of JSB pigs remains moderate, with recorded average total litter size and live-born piglets per litter of 10.79 and 10.20, respectively [12], necessitating further genetic improvement.

In this study, we used six pig populations that differ in litter size (based on their reported total born number) as a comparative framework: a Large White line with high litter size (> 16, LWH), cryopreserved Large White samples from 1977 (LW1977), dam-selected (LWD) and sire-selected (LWS) lines derived from LW1977, Berkshire (BKS), and Jishen Black pigs (JSB). The genome-wide selection-signatures scans were performed between population pairs. Candidate genes were defined as those identified consistently across all high‑ vs. low‑litter‑size comparisons (LWH vs. BKS, LWH vs. JSB, LWD vs. LW1977, and LWD vs. LWS), and further filtered by excluding genes that were also detected in the low‑litter‑size pair comparison (BKS vs. JSB). The resulting set of genes was identified as putative candidates associated with litter size and references for further functional validation study in Jishen Black pigs.

## Materials and methods

###  Resequencing of samples and detection of variants

The whole-genome resequencing (WGS) data were obtained from 19 Jishen Black (JSB) pigs and merged with publicly available WGS from 91 individuals (Table S1). The 110 animals spanning different litter-size gradients were stratified into six groups according to reproduction performance and breeding history: high-litter-size Large White (LWH, *n* = 34), which has phenotype records of 16 total piglets born [13]; dam (LWD, *n* = 12) and sire (LWS, *n* = 13) French Large White lines, which diverged in 1995 and were selected under partly different objectives, and cryopreserved samples from 1977 prior to the divergence (LW1977, *n* = 9) [14]; Berkshire (BKS, *n* = 23), which is low-fecundity with 9 piglets born [15–17]; and Jishen Black pigs (JSB, *n* = 19), a crossbred breed which sows farrowed with average litter size of 10.79 [12], and the record of JSB is slightly higher than the actual value. Information on the selected samples can be found in the Supplementary Materials (Table S1), and no definite direct kinship was identified within the JSB. It should be noted that the limited sample size in certain populations (e.g. LW1977, LWD and LWS) may result in unavoidable bias in the following allele-frequency estimates and haplotype-based statistics.

Raw reads from both newly generated and publicly available datasets were processed using the same pipeline. The pre-processing was performed using Fastp (v 0.24.0) [18] under default settings to discard low-quality sequences, producing high-quality clean reads. These clean reads were aligned to the Sscrofa11.1 reference genome (NCBI) using BWA-MEM (v 0.7.18-r1243-dirty) [19]. Alignment metrics were computed with SAMtools (v 1.21) [20], after which duplicate reads were removed and coordinate-sorted. Single-sample gVCFs were generated by the HaplotypeCaller module of GATK (v 4.6.1.0) [21] pipeline, and subsequently consolidated into a multi-sample VCF using GenotypeGVCFs. At this stage, all individual data were consolidated into a single file, which served as the foundation for all subsequent analyses.

### SNP and indel screening

Variant quality refinement was performed with SelectVariants of GATK pipeline. Stringent hard-filtering thresholds were applied to minimize false positives. The filtering criteria for SNPs were QD < 2.0, QUAL < 30.0, MQ < 40.0, SOR > 3.0, FS > 60.0, MQRankSum < − 12.5, ReadPosRankSum < − 8.0; and the filtering criteria for Indels were QD < 2.0, QUAL < 30.0, MQ < 40.0, FS > 200.0, ReadPosRankSum < − 20.0. Passing variants were functionally annotated with ANNOVAR against the Sscrofa11.1 reference genome, assigning each SNP and Indel to genomic categories (exon, 5′/3′ UTR, intron, splice site, upstream, downstream, intergenic). Exonic SNPs were further subclassified as synonymous or non-synonymous. Following these processing steps, we retained a total of 39,234,894 SNPs.

### Phylogenetic and population genetic analysis

To resolve phylogenetic relationships among populations divergent in reproduction performance, high-quality autosomal biallelic SNPs were subjected to additional quality control (QC) in a single VCF file: minor allele frequency (MAF) < 0.01, Hardy–Weinberg equilibrium (HWE) *p* < 0.001, and individual call rate < 90% were excluded, and 19,236,387 SNPs were retained after QC. Linkage-disequilibrium (LD) pruning was executed by PLINK (v1.90) [22] option “--indep-pairwise 50 5 0.2”, retaining 1,192,623 independent SNPs. A neighbour-joining (NJ) tree with 200 bootstrap replicates was constructed with TreeBeST and visualised in iTOL (v6) (Interactive Tree Of Life) [23]. Population structure was inferred with ADMIXTURE (v1.3.0) [24]. Cross-validation error was assessed for K = 2–7 to determine the optimal number of ancestral components. Principal-component analysis (PCA) was performed with PLINK (v1.9) [22]. Linkage-disequilibrium (LD) block dimensions for candidate genes and associated genomic regions were quantified with PopLDdecay (v3.43) [25]. Finally, the effective population size (*Ne*) was calculated using SNeP (v1.1) [26].

### Selection signature analysis

**Fig. 1:**
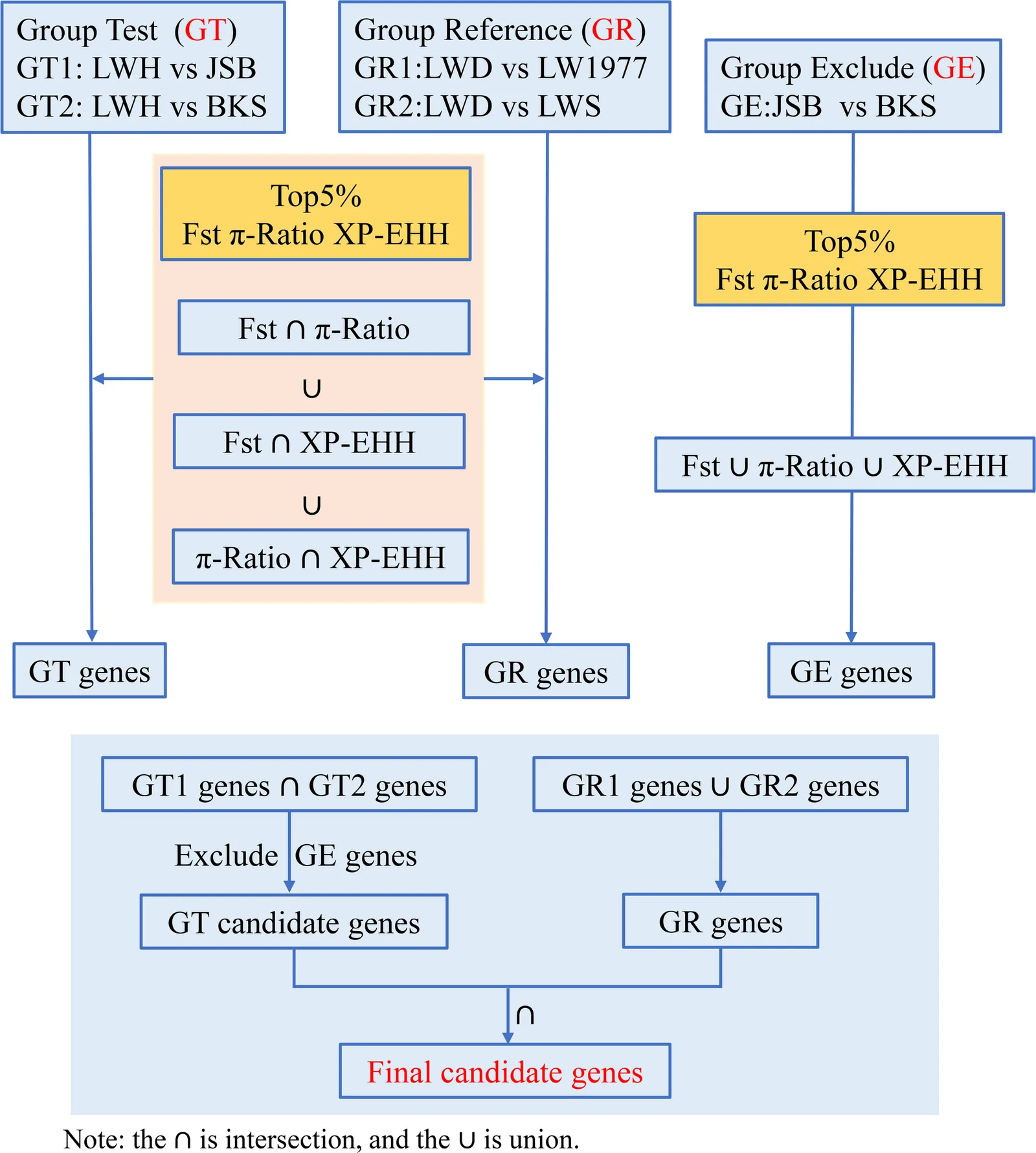
Schematic diagram of the selection-signature scanning strategy

The analytical workflow is illustrated in Fig. 1. Specifically, three genome-wide selection-signature scanning methods were applied to groups differing in litter size levels: the population fixation index (*Fst*), the ratio of nucleotide diversity (π-Ratio), and the cross-population extended haplotype homozygosity (XP-EHH). *Fst* and π-Ratio were calculated with VCFtools v0.1.13 [27] in 50 kb sliding windows with a step size of 10 kb; XP-EHH was computed using selscan [28] with the same window settings, excluding windows containing fewer than 10 SNPs, and normalized the XP-EHH results using the “xpehh2slidingWindow.py” script. To prevent undefined ratios and statistical instability caused by near-zero denominators in π-Ratio calculations [29], genomic windows with π < 1 × 10⁻⁶ were filtered out. This threshold, approaching machine precision, ensures computational robustness while removing biologically negligible variation [30, 31].

Pairwise selection signature scans were conducted across six populations assigned into three analytical groups: Group Test (GT), comparison between LWH and JSB (GT1), and between LWH and BKS (GT2); Group Reference (GR), comparison between LWD and LW1977 (GR1), and between LWD and LWS (GR2); and Group Exclude (GE), a comparison between JSB and BKS. Since the difference in litter size between JSB and BKS is relatively small, numerous signals unrelated to reproduction may arise. Therefore, we excluded genes under selection in JSB and BKS from our results.

For the GT and GR groups, π-Ratio and XP-EHH values were calculated by designating the higher-litter-size level population as the target and the lower-litter-size level population as the reference (e.g., LWH/JSB). Only genomic regions with positive XP-EHH scores were considered, indicating selection in the respective target populations. To balance detection sensitivity and specificity, thereby capturing candidate genes with comparatively weak selection signatures without excessive false positives, the candidate genes under selection were identified as those ranking in the top 5% in at least two of the three scanning methods, following previous studies [32–35]. In the GE group, both positive and negative XP-EHH values were considered, with mean detection values used to define window boundaries. Genes within the top 5% of any method in the GE group were retained for subsequent analysis. To assess the credibility of the identified candidate genes, we calculated the Population Branch Statistic (PBS) from pairwise *Fst* values. LWH was specified as the test population, with JSB and BKS serving as reference populations, thereby allowing us to quantify population-specific divergence at candidate regions (significant windows) consistent with selection.

### Gene annotation and functional inference

Gene annotation files were downloaded from Ensembl BioMart [36] (downloaded on 29 December 2025) to identify genes within selection regions. Any gene whose entire coding sequence fall within a selected window was annotated as a candidate gene, and when multiple genes overlapped a single window, all overlapping genes were retained as candidates. The overlapping genes annotated in both GT1 and GT2, after excluding those found in the GE, were defined as GT candidate genes. Genes annotated in either GR1 or GR2 were combined to generate GR candidate genes. The intersection of GT and GR candidate genes was subjected for subsequent filtering: only genes with unambiguous gene symbols were retained as candidates, those annotated solely with Ensembl IDs were excluded. Finally, we obtained a final set of candidate genes for following in-depth analyses.

To evaluate the relevance of these genes to litter size, we queried PigBiobank [37] and ISwine [38] (accessed on 30 December 2025) to assess their potential as litter-related genes. Functional enrichment analyses were then performed using DAVID [39], incorporating GO and KEGG terms with significance determined at *p* < 0.05 after Benjamini–Hochberg correction. For these final candidate genes, transcript expression data (TPM values) across 34 tissues were downloaded from PigGTEx [40] (downloaded on 18 December 2025) to generate tissue-specific expression profiles.

## Results

### Whole-genome resequencing and variant detection

**Fig. 2:**
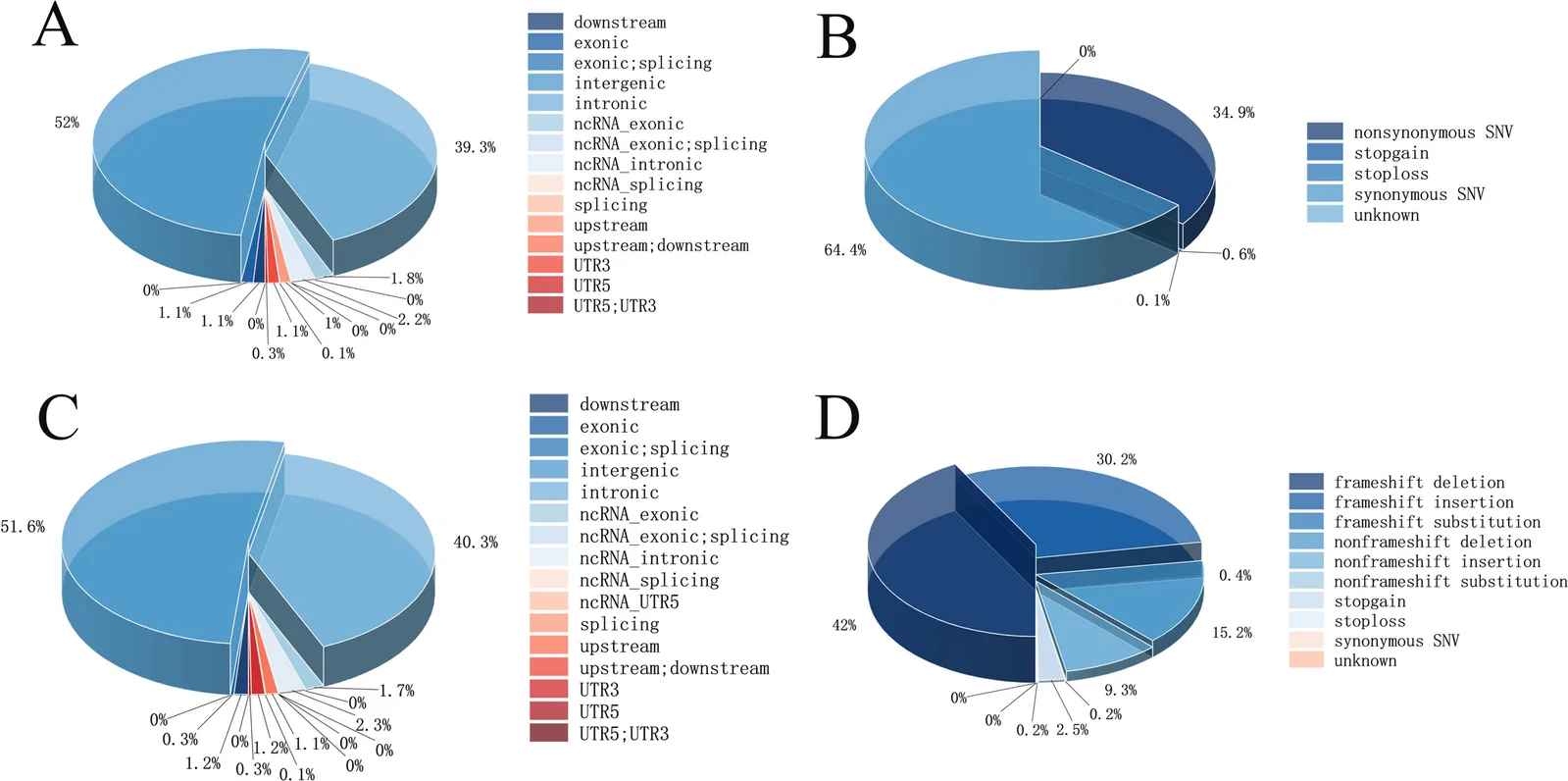
ANNOVAR functional classification of sequence variants identified in 110 resequenced pigs. **A** Genome-wide distribution of all SNPs. **B** Exonic subset of SNPs. **C** Genome-wide distribution of all INDELs. **D** Exonic subset of INDELs

The alignment of our selected samples to the reference genome achieved mapping rates exceeding 97%, with the proportion of correctly mapped paired-end reads generally exceeding 87% (Table S1), collectively indicating robust sequencing quality. A uniform bioinformatics pipeline yielded 39,234,894 SNPs and 8,468,891 indels. ANNOVAR annotation showed that 51.98% of SNPs reside in intergenic regions and 39.32% in introns; and 1.14% map to coding sequences, comprising 155,830 non-synonymous and 287,420 synonymous changes (Fig. 2A and B). A similar distribution was observed for indels, with 51.57% intergenic and 40.27% intronic; and 0.32% are located within exons, including only seven synonymous indels (Fig. 2C and D).

### Genetic diversity and population structure

**Fig. 3:**
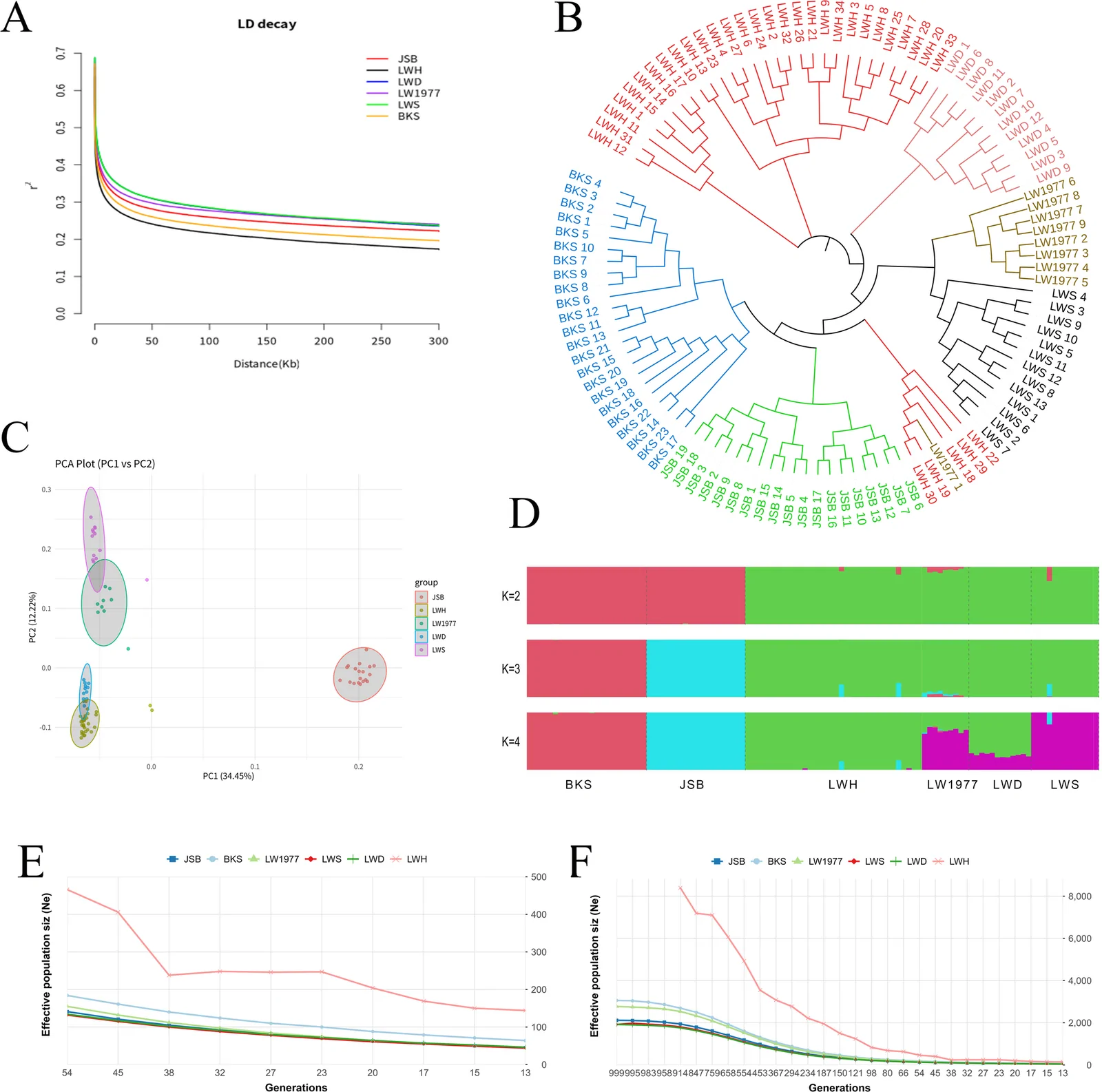
Population genetic characterization of 110 pigs. JSB, Jishen Black pigs; BKS, Berkshire; LW1977, unselected 1977 Large White pigs; LWD, dam-selected Large White pigs; LWS, sire-selected Large White pigs; LWH, high litter size Large White pigs. **A** Genome-wide linkage-disequilibrium (LD) decay. **B** Neighbour-joining (NJ) tree reconstructed from genome-wide SNPs. **C** The PCA plot of populations. **D** Population genetic structure of the 110 individuals for K ranged from 2 to 4. **E** Effective population size (*Ne)* values for 5–50 generations. **F** *Ne* values for 5–1000 generations

Linkage-disequilibrium (LD) landscapes differed among the six populations: at 300 kb physical distance, the LW1977 retained the highest LD (r² ≈ 0.24), whereas the LWH showed the most rapid LD decay, with r² falling to 0.16. The Jishen Black pig, a locally developed composite breed, exhibits strong genome-wide linkage disequilibrium and consequently attenuated LD decay (r² ≈ 0.22) (Fig. 3A, Table S2), reflecting its small effective population size, limited recombination history, and lack of long-term and effective artificial selection.

A genome-wide neighbour-joining (NJ) tree constructed from the 110 individuals resolved six monophyletic clades consistent with breed identity and selection lineage (Fig. 3B). Principal component analysis (PCA) results showed that excluding BKS, PC1 (34.45%) captured the east-west genetic background differentiation, while PC2 (12.22%) additionally captured genetic structure. Visual inspection showed a gradual distribution pattern along PC2. Although this spatial pattern appeared to correlate with litter size variation (Fig. 3C, Fig.S1, Fig.S2), such a potential association remains unvalidated by statistical analysis. Model-based clustering (ADMIXTURE) analysis achieved the lowest cross-validation error at K = 3, clearly separating LWH, LW1977, BKS and JSB. At K = 4, LW1977-derived LWS and LWD lines were further distinguished, revealing signatures of directional selection (Fig. 3D). JSB and BKS clustered together at K = 2, but were fully separated at K = 3. In the PCA, JSB and BKS are proximate along PC1, but JSB is closer to the Large White pig group along PC2 (PC1 = 9.74%, PC2 = 7.18%) (Fig.S3). Effective population size (*Ne*) estimates supported these results (Fig. 3E and F, Table S3). All populations exhibited similar historical declines except LWH. The JSB experienced a 68% reduction, from *Ne* = 141 to *Ne* = 45, indicating that long-term artificial selection and closed breeding substantially reduced its genetic diversity-consistent with its slower LD decay.

### Genome-wide selection-signature scanning

**Fig. 4:**
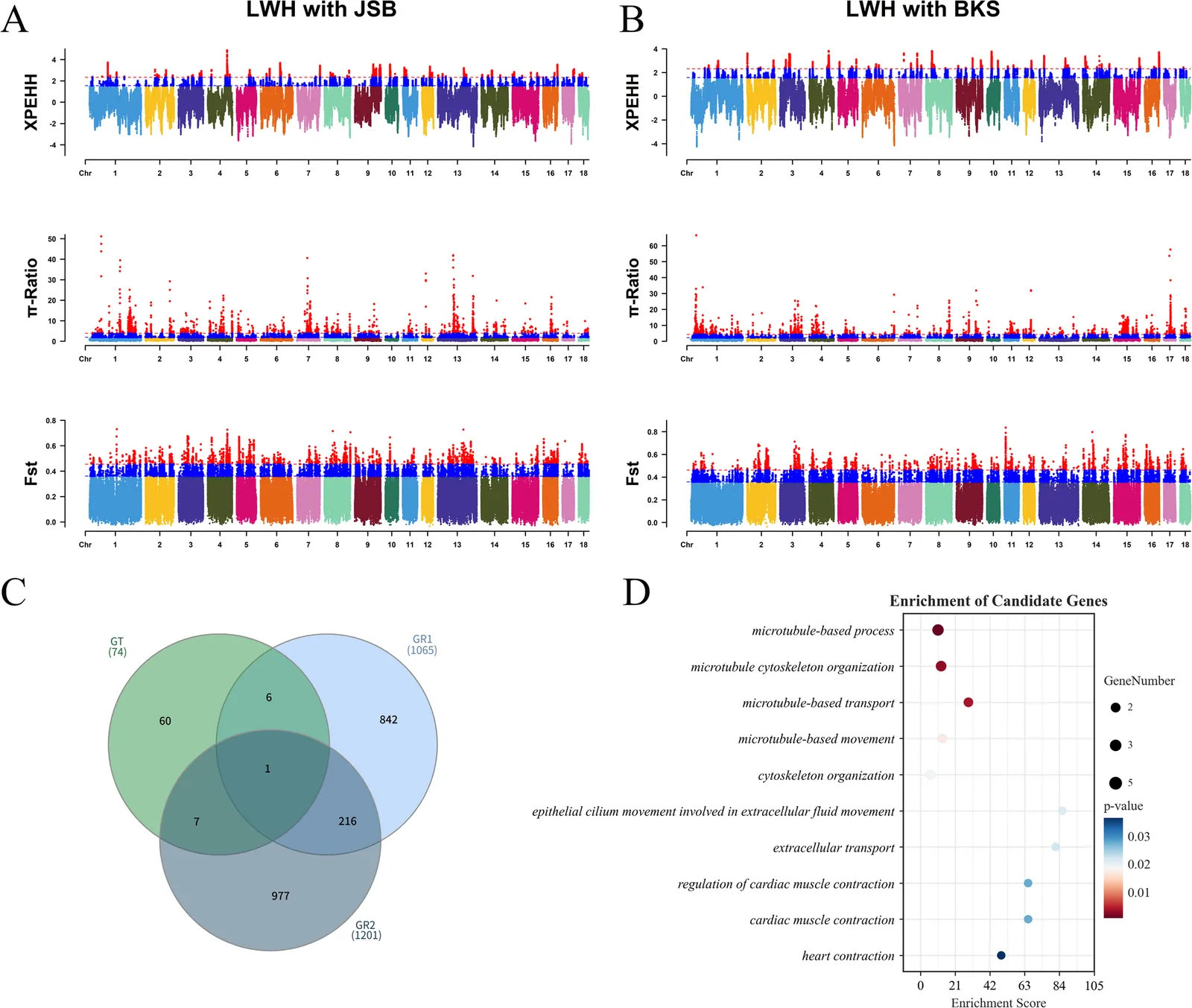
Candidate gene identification. LWH: High litter size Large white; JSB: Jishen Black pigs; BKS: Berkshire; GT: Group test; GR: Group reference. **A** Selection-signature scanning results for JSB and LWH. **B** Selection-signature scanning results for LWH and BKS. **C** Venn diagram of GT, GR1 and GR2 cross genes. **D** Candidate gene enrichment analysis bubble chart

We performed genome-wide selection scanning across the six populations following the workflow as demonstrated in Fig. 1. In total, 1177 genes were identified by at least two methods in GT1 (Fig. 4A, Table S4); 1391 genes in GT2 (Fig. 4B, Table S5); 1065 genes in GR1 (Fig.S4, Table S6); 1203 genes in GR2 (Fig.S5, Table S7); and 1473 genes in GE (Fig.S6, Table S8). Intersecting GT1 and GT2 genes produced 166 candidates (Table S9), and after excluding GE-derived genes, 74 genes remained (Table S11) as GT candidate genes. The union of GR1 and GR2 yielded 217 genes as GR candidate genes (Table S10). After further intersecting the GT with GR candidates, we identified 14 genes. These genes include *PEX14*, ENSSSCG00000054324, ENSSSCG00000042623, *CDK15*, *KCNQ1*, *SPAG17*, *TTF2*, *CD101*, *CASQ2*, *VANGL1*, *ARID5B*, *KLHL32*, *EML1*, and *NAV1*. After excluding two additional genes annotated solely with Ensembl ID, we finally obtained 12 genes as the most possible candidates for litter size. Notably, *KCNQ1* was consistently detected in all high-low fertility contrasting pairs, GT1, GT2, GR1 and GR2 (Fig. 4C), underscoring its robustness as a key candidate. Population Branch Statistic (PBS) analysis provided additional support for the robustness of the candidate gene set (Table S12). All selected genes contained windows with PBS > 0.18, and the highest window-level PBS reached 0.62 (thresholds were 0.208 for the top 1% and 0.147 for the top 5%). The magnitude of these elevated PBS values is therefore indicative of pronounced population-specific differentiation and can be interpreted as evidence consistent with selection acting on these regions.

### Gene enrichment analysis and functional annotation

Functional enrichment analysis revealed significant overrepresentation in microtubule-related biological processes, including microtubule-based process (*p* = 0.000579), microtubule cytoskeleton organization (*p* = 0.00256) and microtubule-based transport (*p* = 0.00378) (Fig. 4D, Table S13). The genes implicated in these pathways included *EML1*, *VANGL1*, *NAV1*, *PEX14*, and *SPAG17*. Examination of GO and KEGG functional terms (Table S14) demonstrated that these genes exhibit pleiotropic effects, participating in multiple biological processes such as mitotic cell cycle regulation, cell population proliferation, and cell cycle progression.

Expression profiling from the ISwine databases revealed tissue-specific patterns: *PEX14* and *KCNQ1* are highly expressed in ovaries, *CASQ2* in placenta and ovarian granulosa cells, *NAV1* in granulosa cells, *CDK15* in sperm, *SPAG17* in testis, and *KLHL32* in oocytes. QTX (Quantitative Trait Loci/Gene/Nucleotide) mapping linked *PEX14* and *CDK15* to litter size, and *EML1* and *NAV1* to total litter weight. The PheWAS (phenome-wide association study) results from PigBiobank database further demonstrated the association of *CDK15*, *EML1*, and *NAV1* with litter size; *CDK15*, *CASQ2*, and *VANGL1* with the number of live-born piglets; *PEX14*, *TTF2*, *CD101*, and *ARID5B* with total weight of live-born piglets; and *KCNQ1* and *KLHL32* with number of healthy piglets.

In total, 12 genes (*KCNQ1*, *PEX14*, *CDK15*, *SPAG17*, *TTF2*, *CD101*, *CASQ2*, *VANGL1*, *ARID5B*, *KLHL32*, *EML1*, and *NAV1*) were identified as strong candidates influencing litter size, with *KCNQ1* emerging as the top candidate.

### Analysis of *KCNQ1*

**Fig. 5:**
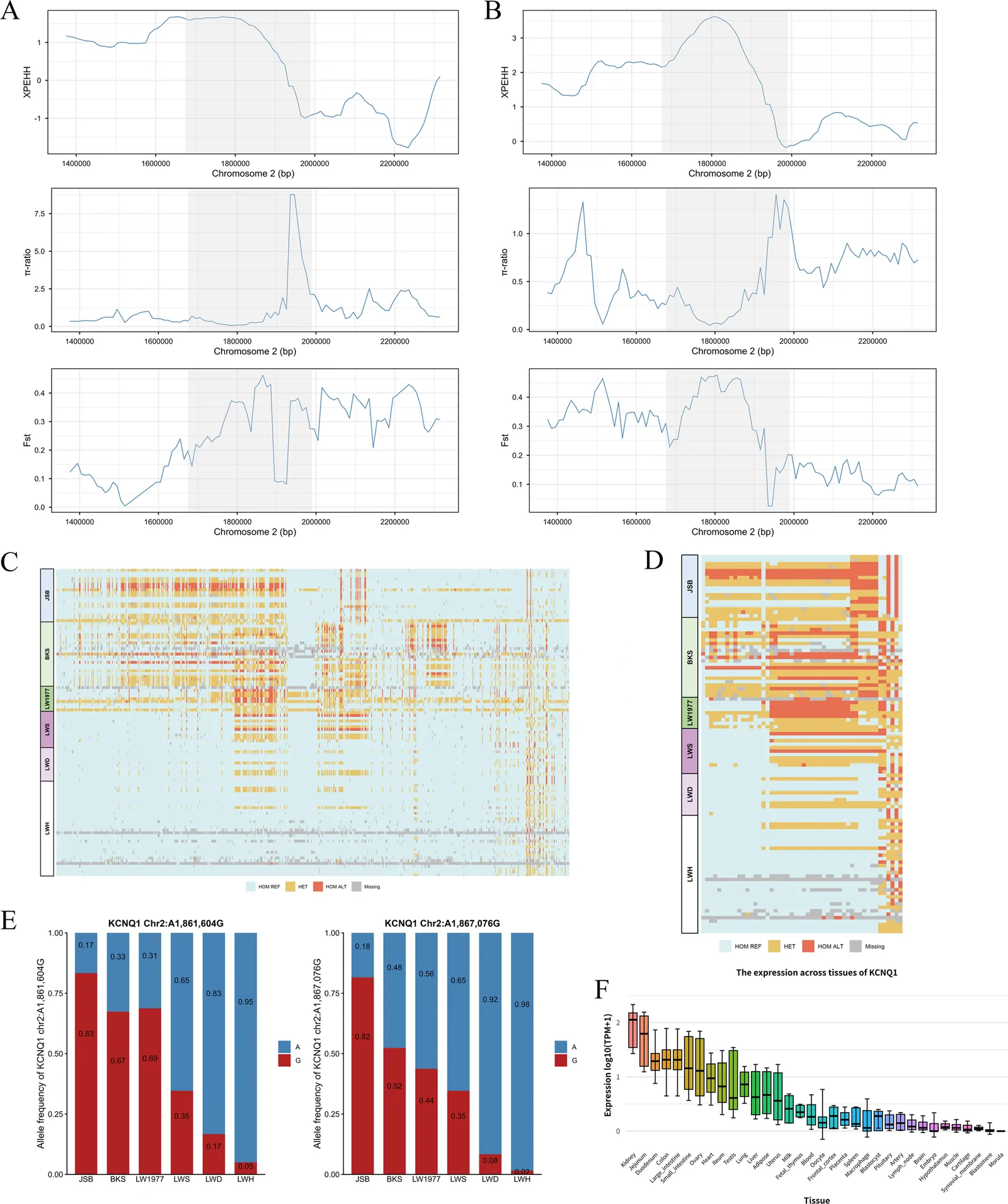
Analysis of *KCNQ1*. **A** The *KCNQ1* region exhibits parallel sweep signals in LWH and JSB. **B** The *KCNQ1* region exhibits parallel sweep signals in LWH and BKS. **C** Genotype heat-maps of loci across *KCNQ1*; HOM ALT = homozygous alternate (1/1), HET = heterozygous (0/1 or 1/0), HOM REF = homozygous reference (0/0). **D** Genotype heat-maps of the top 50 significant loci across *KCNQ1.* **E** Allele-frequency distribution of the *KCNQ1* chr2:1,861,604 and chr2:1,867,076 variant among the six populations. **F** The tissue expressions of *KCNQ1*

Detailed investigation of *KCNQ1* revealed strong selection signals across *Fst*, π-Ratio, and XP-EHH analyses in GT1 (LWH vs. JSB) and GT2 (LWH vs. BKS) (Fig. 5A and B). SNP genotyping within the *KCNQ1* region indicated substantial allelic variation across populations (Fig. 5C). Allele frequency differences were tested using Chi-squared (*X*^*2*^) tests and Fisher’s exact tests, with Benjamini–Hochberg correction applied. The top 50 loci based on adjusted *p*-values were visualized (Fig. 5D). Two intronic SNPs (chr2:1,861,604 and chr2:1,867,076) showed allele frequency gradients consistent with litter-size stratification (Fig. 5E, Table S14). At chr2:A1,861,604G, the ref: alt (A: G) allele counts were 6:30, 15:31, 5:11, 19:7, 20:4, and 55:5 in JSB, BKS, LW1977, LWS, LWD, and LWH, respectively; corresponding counts at chr2:A1,867,076G (A: G) were 7:31, 20:22, 9:7, 17:9, 22:2, and 63:1. The expression of *KCNQ1* was also higher in ovarian tissue (Fig. 5F). These results strongly suggest the possible functions of *KCNQ1* in proliferation, though its actual biological role requires further functional validation.

## Discussion

The Jishen Black pig (JSB), derived from Large White and Beijing Black pigs, maintains a mean litter size of 10.79 [12], which remains considerably lower than that of highly selected Large White herds. Through analysis of the whole-genome resequencing of populations spanning a broad range of litter-size phenotypes, including selected and unselected Large White populations, we dissected the genetic architecture underlying reproduction performance using an integrated framework combining population genetics and selection signature analyses.

To increase robustness and reduce method-specific bias, we integrated three complementary statistics for detecting selective sweeps: *Fst*, π-Ratio, and XP-EHH. *Fst* and π-Ratio are based on site-frequency spectrum (SFS) information and primarily capture allele-frequency differentiation and changes in within-population genetic diversity, respectively. These statistics are sensitive to shifts in allele frequencies caused by selection but may also reflect demographic processes. In contrast, XP-EHH is a haplotype-based method relying on linkage disequilibrium (LD) patterns and detects extended haplotype homozygosity differences between populations. It is particularly powerful for identifying recent or nearly fixed selective sweeps in a target population. By combining SFS-based (*Fst* and π-Ratio) and LD-based (XP-EHH) approaches, we aimed to enhance detection sensitivity while increasing specificity. Candidate regions were defined as genomic windows simultaneously supported by at least two independent statistics, thereby reducing false positives that might arise from reliance on a single method.

Pairwise comparisons between genetically differentiated populations may capture signals shaped by demographic history, breed formation, or genetic drift. To mitigate this confounding effect, we implemented a multi-population comparative framework structured into three analytical modules, including GT (Gradient Test group) for contrasts along the litter-size gradient; GR (Reference group) for historical and selection-line contrasts; GE (Genetic Exclusion group): for outgroup comparison (JSB vs. BKS) to filter out other confounding signals. Selection signals detected in GE were excluded from GT results to minimize the influence of breed-specific demographic history. This hierarchical design allowed us to focus on genomic regions consistently associated with fecundity stratification rather than simple breed divergence.

Our findings delineate clear genomic footprints of artificial selection for reproductive traits within Large White sublines. The 1977 Large White population and its derived sire/dam lines shared comparable LD patterns and a common gene pool. In contrast, the high-fertility LWH line displayed accelerated LD decay, reflecting intense selection for prolificacy. The moderate LD decay in JSB likely results from its mixed ancestry and modest artificial selection pressure. ADMIXTURE analysis at K = 2 indicated a closer genetic relationship between JSB and BKS than between JSB and the Large White sublines. This pattern can be explained from several complementary perspectives. The paternal ancestral lineage of JSB, the Beijing Black pig, is a composite breeds with Berkshire genetic background [11], which likely contributes to the observed genetic affinity between JSB and BKS. Moreover, Berkshire pigs, although originally of European origin, have undergone extensive breeding and selection in East Asian countries such as Japan and Korea, resulting in detectable introgression of Asian-derived haplotypes and partial genomic convergence with Asian breeds [41]. In addition, ADMIXTURE tends to capture the dominant axis of genetic differentiation when sample sizes are imbalanced [24]. Because the combined sample size of the three Large White sublines far exceeded that of BKS or JSB, the algorithm extracted the major east-west ancestry contrast as the first principal component, assigning the Large White lines to one cluster and merging JSB and BKS into another. This outcome is consistent with PCA results, where PC1 primarily reflects geographical differentiation, while PC2 represents finer population structure, corroborating earlier findings in domestic pig population studies [42]. Furthermore, the selection signal scans detected a greater number of differentiated genome regions and associated genes between JSB and BKS than between any other pair of populations (Table S8), indicating a relatively larger genetic distance between these two breeds despite their shared ancestry signals.

The integrating multiple selection-scan methods (*Fst*, XP-EHH, π-Ratio) are widely regarded as an effective strategy to capture diverse genomic footprints of artificial selection [43]. By contrasting high- and low-fertility populations while controlling for unrelated differentiation (removing the selected genes identified by JSB vs. BKS), we refined signals potentially associated with reproduction performance. Many genes identified in JSB vs. BKS are associated with growth and carcass traits (e.g., *ABCD4*, *IGF2R*, *HMGA1*, *PRKAG3*, *ORC3*, *RELN*, *IGF2BP3*) [44–51] and were therefore excluded, enhancing the prioritization of candidates potentially related to reproduction traits.

Among the identified loci, several genes, including *KCNQ1*,* PEX14*,* CDK15*,* SPAG17*,* TTF2*,* and VANGL1*, have documented roles in reproductive processes. *KCNQ1*, exclusively expressed in porcine granulosa cells and antral follicles, has been functionally implicated in ovarian morphogenesis [52] and follicular development [53], and associated with fertility in cattle [54]. *PEX14* marks peroxisomes across follicular developmental stages throughout all oestrus cycles in all ovarian cell types [55], and has also been detected in oocytes at all oestrous cycle phases [56]. *CDK15* encodes a kinase involved in cell cycle regulation [57], analogous to *CDK6*, which mediates follicle transition [58]. *SPAG17* influences germ cell differentiation and fertility [59], influences litter size in sheep [60], and ducks [61]. *TTF2* regulates thyroid-related pathways impacting parathyroid gland development [62], and hence follicular development [63, 64] and oocyte quality [65]. *CD101* [66] and VANGL1 [60, 67] contribute to follicular development and embryo implantation. *ARID5B*, *KLHL32* and *EML1* exhibit reproductive expression patterns and are linked to reproduction in geese, Taihu pig and goats [68–71]. The enriched microtubule-related pathways identified were important in oocyte maturation and ovulation [72]. These established associations support the potential biological plausibility, but their functional role in litter-size variation of pigs remains unverified. Among all candidates, *KCNQ1* is the most robust candidate gene prioritized in this study. Its recurrent detection across independent analyses and allele frequency gradients consistent with reproduction performance support its status as a strong candidate. Nevertheless, this evidence is correlational, and functional validation is required.

It should be noted that our selection signature analysis operates under the assumption that inter-population variation in litter size predominantly reflects genomic divergence. Owing to the unavailability of individual phenotypic records we utilized litter size stratification level based on total number born, which precludes exclusion of phenotypic outliers within low litter size level populations. PCA is based entirely on genomic variation and therefore cannot be used to infer phenotypic differentiation. Furthermore, given that swine reproduction traits are under polygenic control with modest effect sizes, our scan can only capture a fraction of the underlying genetic architecture. Consequently, these findings should be interpreted as probabilistic outcomes, and all candidate genes require rigorous experimental validation.

It should be emphasised that the selection-signature scanning strategy employed in this study is predicated upon the premise that differences in reproduction efficiency primarily stem from allele differentiation driven by artificial selection. And the population-based selection scans identify associations rather than direct causation. Potential confounding from background selection or linkage effects cannot be completely excluded. Therefore, these findings should be regarded as strong candidate hypotheses rather than confirmed causal loci. Functional validation through independent populations, transcriptomics, and gene-editing experiments will be essential to confirm causal relationships and elucidate the mechanisms underlying reproduction efficiency.

## Conclusion

This study performed a genome-wide characterization of genomic differentiation among pig populations with divergent reproductive performance based on whole-genome resequencing data of 110 individuals. By integrating *Fst*, π-Ratio, and XP-EHH analyses, we identified 12 candidate genes showing signatures of population-level selection. Among these, *KCNQ1* exhibited the most prominent selection signals and plausible biological relevance to reproductive traits. Two intronic SNPs within *KCNQ1* (chr2:A1,861,604G and chr2:A1,867,076G) displayed allele frequency stratification across populations, which descriptively coincided with the phenotypic divergence in litter size.

Overall, this work provides a set of candidate genomic regions and genes derived from population-level selection signature analyses for future studies on porcine reproductive traits. It should be noted that the present analyses were based on population-averaged phenotypic information without individual-level genotype–phenotype association validation. The detected selection signals may be confounded by population structure, demographic history, breed formation, and selection on other complex traits. Therefore, further validation using independent populations and functional experiments is required to clarify their potential roles and causal relationships with litter size.

## Supplementary Information

Supplementary Material 1.

## Data Availability

The data used in this study can be obtained from the NCBI and CNCB databases (BioProject numbers can be found in Table S1).
